# MicroRNAs: A Link between Mammary Gland Development and Breast Cancer

**DOI:** 10.3390/ijms232415978

**Published:** 2022-12-15

**Authors:** Diana Wu, Lilian U. Thompson, Elena M. Comelli

**Affiliations:** 1 Department of Nutritional Sciences, University of Toronto, Toronto, ON M5S 1A8, Canada; 2 Joannah and Brian Lawson Centre for Child Nutrition, University of Toronto, Toronto, ON M5S 1A8, Canada

**Keywords:** mammary gland, microRNA, development, breast cancer

## Abstract

Breast cancer is among the most common cancers in women, second to skin cancer. Mammary gland development can influence breast cancer development in later life. Processes such as proliferation, invasion, and migration during mammary gland development can often mirror processes found in breast cancer. MicroRNAs (miRNAs), small, non-coding RNAs, can repress post-transcriptional RNA expression and can regulate up to 80% of all genes. Expression of miRNAs play a key role in mammary gland development, and aberrant expression can initiate or promote breast cancer. Here, we review the role of miRNAs in mammary development and breast cancer, and potential parallel roles. A total of 32 miRNAs were found to be expressed in both mammary gland development and breast cancer. These miRNAs are involved in proliferation, metastasis, invasion, and apoptosis in both processes. Some miRNAs were found to have contradictory roles, possibly due to their ability to target many genes at once. Investigation of miRNAs and their role in mammary gland development may inform about their role in breast cancer. In particular, by studying miRNA in development, mechanisms and potential targets for breast cancer treatment may be elucidated.

## 1. Introduction

Breast cancer is among the most common cancers in women, second to skin cancer [1]. Treatment of breast cancer is complex and often depends on the subtype, as developed by Perou et al. [2]. Currently, breast cancer subtypes have evolved to include genomic, proliferative, and immune cell markers [3]. Early breast cancer detection and prevention is commonly associated with BRCA1 or BRCA2 mutations [4], but other structures in the mammary gland may have predictive value as well. For instance, during puberty, terminal end bud (TEB) structures develop, which are the most common sites for tumorigenesis. In later life, these structures differentiate into alveolar buds (AB), which have a reduced risk of de novo tumorigenesis. It has been shown that the number of TEB structures in early life is modifiable, for example via dietary interventions, leading to reduced breast cancer risk later in life [5]. Furthermore, processes critical to normal mammary gland function such as apoptosis, proliferation, and invasion are often altered, leading to breast cancer formation. In fact, a subset of microRNAs (miRNAs) with expression varying through stages of development (juvenile, puberty, mature virgin, gestation, lactation, early involution, and late involution) were also found to be associated with the luminal A breast cancer subtype [6]. This suggests that parallel roles for oncogenic and anti-oncogenic miRNAs may exist during development and during breast cancer. MiRNAs are short, non-coding RNAs, and are required for normal development across species. In the mammary gland, aberrant expression of miRNAs can alter critical functional and developmental processes leading to the development of breast cancer [7]. Therefore, miRNA expression in the mammary gland is a promising clinical biomarker. MiRNAs are also a promising therapeutic target for breast cancer. Investigation of the role of miRNAs in mammary gland development can help to improve our understanding of which miRNAs affect apoptosis, proliferation, invasion, and angiogenesis, and how these miRNAs may serve an oncogenic or tumour suppressive role in breast cancer [7]. It may also provide important insight for preventative strategies. In this review, we provide an overview of the stages of mammary gland development, the stages and characteristics of breast cancer, and discuss miRNAs that may affect both processes.

## 2. Mammary Gland Development

**Figure 1:**
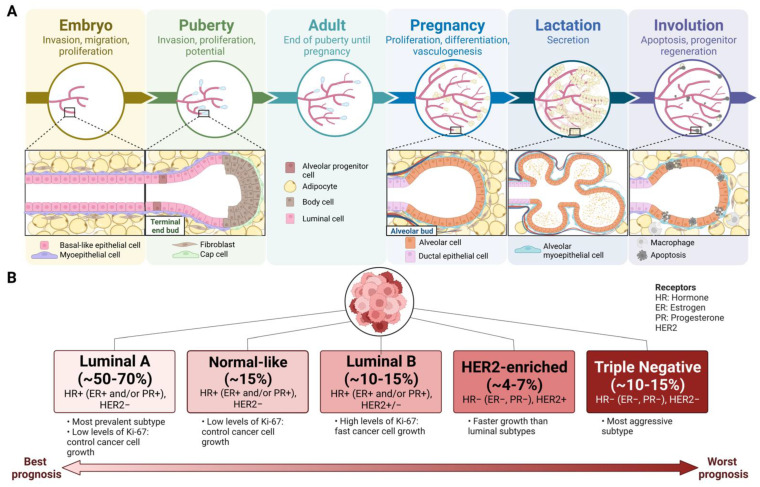
(**A**), A timeline of mammary gland development through six stages and (**B**), Intrinsic breast cancer subtypes, markers, and characteristics from best prognosis to worst prognosis. Cancer type incidence are as per [1,9] Created with BioRender.com.

Mammary gland development occurs in five stages: embryo, puberty, pregnancy, parturition, and involution [8] (Figure 1A). Below, we provide a brief synopsis of this process, with reference to the time points that are relevant for miRNA regulation, as discussed in Section 5.

Embryo: The mammary gland is separated into the ectoderm and mesoderm, with development beginning around day 10, where it appears as an epithelial bud. The ectoderm forms multiple layers which develop into one pair of placodes (five pairs in mice, six pairs in rats) by day 12 [8]. Around day 13, epithelial cell fate begins to be guided by inductive signals from the mesenchyme. This signaling guides the patterning of the placodes and the positioning of extracellular matrix components. At day 14, the epithelium of the placodes expands and invades the preadipocytes, which are thought to emerge from the mesenchyme [10]. The epithelial cells reach these cells and begin branching, leading to an early ductal system. The ductal lumen forms from day 16 to day 18. Finally, the sexual delineation and nipples are formed. During embryogenesis, the epithelial-mesenchymal interaction and transformation plays a critical role in polarization, tissue repair, and formation of the mammary gland.

Puberty: Around 2–3 days after birth, preadipocytes are completely differentiated into adipocytes [11]. Mammary gland growth is largely isometric until puberty (6–8 weeks postnatal in rodents), when mammary growth becomes allometric [8,12]. This growth is triggered by paracrine and endocrine signals from the pituitary gland, hypothalamus, and gonads. These signals include growth factors and hormones, which develop the rudimentary ductal system into a branched ductal system prepared for pregnancy. The ends of preliminary ducts invade the fat pad and differentiate into TEBs. TEBs and connected ducts are composed of myoepithelial cells on the outer layer and a thicker inner layer of luminal epithelial, with a layer of cap cells at the end of the TEB which have multipotent capabilities [13]. The duct is surrounded with stromal cells and adipocytes. Secondary and tertiary branches emerge from the primary ductal branches, leading to a more expansive ductal system. Overall, pubertal mammary gland growth is characterized by increased invasion and proliferation. After puberty, the mammary gland remains relatively quiescent until pregnancy.

Pregnancy: During estrous cycles and pregnancy, the ductal lumen undergoes minor developments of small, sac-like structures that protrude 90° from the ducts, often called AB. Some of these AB elongate to form secondary or tertiary branches. During pregnancy, this branching is increased in response to progesterone and prolactin secretion from the ovaries and pituitary gland, respectively [8]. The mammary gland undergoes ductal and alveolar proliferation with the guidance of progesterone and prolactin. The alveoli continuously expand and divide, with the alveolar epithelial cells invading the remainder of the fat pad such that the alveoli have filled most of where the adipose used to be. The alveoli also form clusters surrounded by blood and secretory vessels, prepared for lactation.

Lactation: Post-pregnancy, serum progesterone levels drop significantly and the number of prolactin receptors on alveolar cells increase [8]. These alveolar cells are polarized and sequester proteins, fats, and nutrients, while lactating. Prolactin levels increase in response to nursing stimuli, and decrease without, with involution beginning as soon as 24 h of no stimuli.

Involution: Involution begins within 24 h of weaning. It occurs in two phases: in the first phase, the mammary gland alveoli undergo apoptosis without an appreciable change in structure [8]. This phase of involution is reversible within 48 h with the resumption of suckling [14]. The second phase of involution is irreversible. After 48 h of weaning, the second phase begins, which involves destruction of the alveolar structures and lactation-competent cells. With the breakdown of the alveolar structures, adipose cells repopulate the mammary gland and the mammary gland structure returns to the pre-pregnancy state.

## 3. Breast Cancer

Breast cancer is diagnosed and treated based on a subtype-classification system that delineates tumours based on genomic, proliferative, and immunological markers. There are five main types of breast cancer: luminal A-like (ER+, PR+, HER2-, low Ki-67), normal-like HER2- (lower ER+, PR+, HER2-, high Ki-67), luminal B-like HER2+ (ER+, PR+, HER2+, high Ki-67), HER2-enriched (ER-, PR-, HER2+, high Ki-67, non-luminal), and triple-negative (ER-, PR-, HER2-, high Ki-67) [4] (Figure 1B). Triple-negative breast cancer can be divided into six categories which determine its proliferative, apoptotic, and invasive characteristics: basal-like 1, basal-like 2, immunomodulatory, mesenchymal, mesenchymal stem cell-like, and luminal androgen receptor. Out of these, luminal A-like breast cancer is the most commonly found in women and has the highest survival rate [15]. Triple-negative breast cancer has the worst prognosis, with a highly metastatic, aggressive, and invasive phenotype [16]. Several factors are involved in the likelihood of malignant tumour development and metastasis in the mammary gland. Oncogenic factors include the number of undifferentiated structures (particularly TEB) [13], number of estrous cycles [17], mammographic density [18], and early age at menarche [9]. Factors affecting the potential for epithelial-to-mesenchymal transition (EMT) may increase the likelihood of metastasis and expression of breast cancer stem-cell-like properties [19]. Anti-oncogenic factors include early age at pregnancy and factors associated with pregnancy such as high doses of estrogen and progesterone, differentiation of TEB structures, and involution [12].

Breast cancer largely arises in epithelial cells in terminal ductal lobular units [20], or undifferentiated TEB in rodents [21]. The TEB, when in the process of differentiating, also has the potential for carcinogenesis. The differentiation of TEB into AB reduces the potential for the development of malignant tumours. This has been demonstrated with 7,12-dimethylbenz[a]anthracene (DMBA) in vivo [22,23], where administration of DMBA at 20 days of age resulted in death of 36/42 rats. Tumour incidence increased with age until 46 days, after which there was a decrease in malignant tumour incidence and increase in benign tumours.

## 4. MicroRNAs

MiRNAs are short, non-coding 19–22 nucleotide RNAs which post-transcriptionally regulate gene expression [24]. MiRNAs are transcribed by RNA polymerase II in the nucleus, after which Drosha—a ribonuclease type III (RNAse III)—and DGCR8 binds and preprocesses the pre-miRNA. The pre-miRNA (~70 nt long) is transported into the cytoplasm where another RNAse III Dicer cuts pre-miRNA into mature miRNA approximately 22 nt long. This mature miRNA associates with argonaute protein (AGO) to form a RNA-induced silencing complex (RISC) where the miRNA can bind to the 3′ UTR of a mature mRNA, among other possible binding sites, as previously reviewed [24]. One miRNA can target multiple mRNAs, and one mRNA can be regulated by multiple miRNAs, allowing for the coordinated regulation of several mRNA targets and, by extension, proteins and pathways. In development, miRNAs are critical in regulating developmental timing and progenitor cell fate, and these roles have been extensively reviewed by DeVeale et al. [25]. Dysregulation of miRNAs involved in developmental or homeostatic processes can lead to breast cancer; the role of miRNAs involved in breast cancer development, treatment, diagnosis, prognosis, and in exosomes have been previously reviewed [26,27,28]. Here, we discuss miRNAs which can be found in both mammary gland development and breast cancer in the context of functions they may have in common.

## 5. MiRNAs in Mammary Gland Development and Breast Cancer

**Figure 2:**
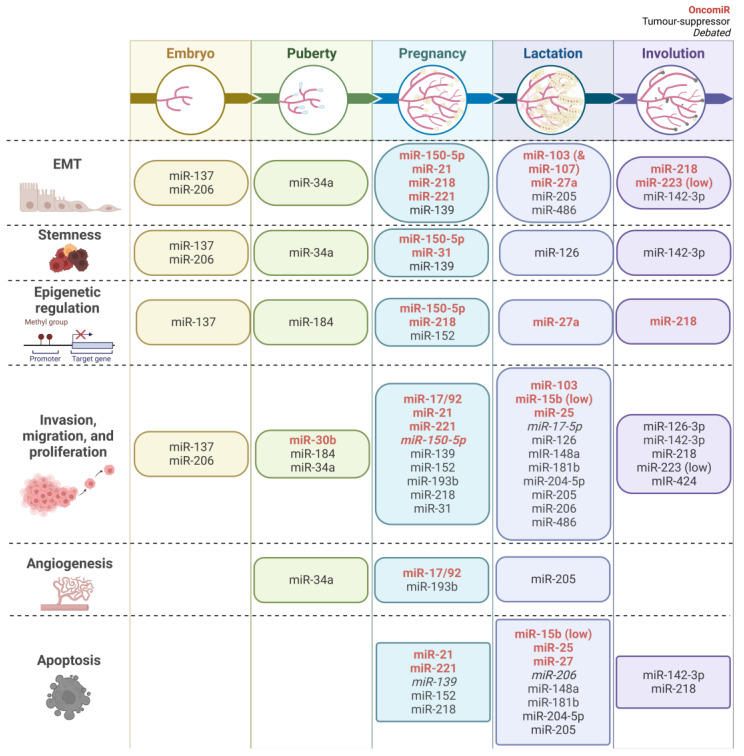
Breakdown of 32 microRNAs found in mammary gland development and breast cancer processes based on their association with their typical stages. Rows correspond to biological mechanisms that can be found in both processes. Columns correspond to stages of mammary gland development. MicroRNAs in red are oncogenic microRNAs. MicroRNAs in black are tumour suppressive in breast cancer. Italicized microRNAs have a debated role in breast cancer. Created with BioRender.com.

This section discusses characteristics common to development and cancer of the breast, such as EMT, stem-cell characteristics, proliferative ability, angiogenesis, apoptosis, and epigenetic regulation. Previous reviews on miRNAs in mammary gland development and breast cancer focus on subsets of developmental stages [29,30]. Here, we seek to provide a comprehensive updated review of miRNAs in mammary gland development and breast cancer. The literature was searched for miRNAs in mammary gland development and breast cancer across species; 32 miRNAs were found to be shared in both processes, as specifically discussed below and illustrated in Figure 2.

### 5.1. EMT

Embryogenesis in the mammary gland is marked by differentiation, migration, and invasion facilitated by a progenitor cell population and epithelial–mesenchymal reciprocity [31]. One hallmark of breast cancer is the reactivation of embryonic programming, particularly with respect to stem-like breast cancer traits and EMT, leading to oncogenesis and metastasis. Embryonic development relies on a delicate interplay between regulatory molecules, such as miRNAs, and expression of genes or transcription factors, and aberrant expression of miRNAs at any stage can dysregulate gene expression leading to breast cancer invasion or metastasis. EMT traits also increase breast cancer chemoresistance by inhibiting apoptosis and increasing chemoresistance-related gene expression [32]. Previous work on embryonic programs in breast cancer has largely focused on parallels in gene-based signatures and pathways [33], and here we will explore this link through miRNA expression. During embryogenesis, miR-137 was found to be highly expressed in the mammary gland, a 30-fold increase compared to surrounding regions [34]. Lentiviral overexpression of miR-137 in ICR mouse embryos led to the thickening of mammary epithelium and inhibition of invasion of the mammary epithelial bud, while MDA-MB-231 tumour formation in vivo was inhibited by miR-137 overexpression [34]. The restriction of mammary bud invasion and tumour formation is consistent with a decreased ability for EMT, where EMT loss may reduce the ability of mammary cells to lose polarity and migrate/invade the underlying fat pad [35]. Indeed, several studies have shown a suppression of invasion, migration, or EMT from miR-137 overexpression [36,37,38,39], leading to reduced metastatic potential and chemoresistance. For example, Lee et al. found that miR-137 was downregulated in triple-negative MDA-MB-231 and Hs578T breast cancer cell lines, and targeted the 3′ UTR of *Del1*, encoding for a protein which is abundantly expressed in plasma of breast cancer patients [38]. MiR-137 has been found to increase protein expression of epithelial marker E-cadherin, and reduce mesenchymal markers N-cadherin and vimentin by modulating *DUSP4*, leading to reduced doxorubicin resistance [37]. The overexpression of miR-137 in triple-negative MDA-MB-231 cells decreased migration and invasion, pointing to a tumour suppressor role of miR-137 in breast cancer. Thus, an embryogenesis-related miRNA is dysregulated in breast cancer EMT. As shown in Table 1, only one other miR (miR-206) that is altered in mammary gland embryogenesis has been studied in breast cancer, highlighting a research gap for further investigation.

### 5.2. Stemness Characteristics

A stem cell population is present in the mammary gland at all stages of development. Multipotent stem cells are present in the embryo, and, as the development stage progresses, the progenitor cell population becomes restricted to bipotent and unipotent progenitors. These progenitor cells are most active in puberty and pregnancy, when there is rapid proliferation and differentiation of ductal structures or alveologenesis. Some “stem cell” genes have been found to be exclusively expressed in the outer cap cell layer of the TEB in puberty, while others are also present in the basal cell population. The multipotent mammary stem cells have a gene signature which resembles claudin-low and basal-like breast cancer types, characterized by increased aggressiveness and metastasis [40]. Due to their plasticity, stem-cell-like breast cancer cells, identified by CD44^+^/CD24^−/low^ markers, have an increased proclivity for invasion, treatment resistance, and cancer recurrence [19].

During embryogenesis, miR-206 was found to be highly expressed in the mesenchyme at day 11.5. At day 13.5, miR-206 expression was reduced in the mesenchymal layers and localized to the mammary fat pad. Overexpression of miR-206 led to severe stunting of mammary bud formation, indicating that miR-206 may abrogate mesenchymal differentiation [41]. MiR-206 has also been found to be highly expressed in pregnancy, indicating it may play a role in restricting lineage/differentiating progenitor cells [42]. In triple-negative breast cancer cell lines, miR-206 mimics reduced the CD44^+^/CD24^−/low^ cell population. In breast cancer stem cells, miR-206 has been found to inhibit proliferation, metastasis, and increase apoptosis [43]. However, miR-206 has also been found to promote MDA-MB-231 and SK-BR-3 in vitro invasion, migration, and proliferation as well as tumour size in vivo [44]. The differences in response may be due to differing cell types or heterogeneity of miRNA targets, and further investigation is necessary.

Female puberty is characterized as a period of ductal growth, invasion, and proliferation, led by differentiation of bipotent and unipotent stem cells. The nulliparous TEB contains a unique progenitor cell population, with alveolar progenitors, basal progenitors, and cap cells able to differentiate into myoepithelial cells [13]. MiRNAs that are enriched/depleted in TEBs or alter TEB morphology may give information as to their role in breast cancer. For example, miR-34a expression has been found to be low in a stem cell (PKH26+ or CD61+/CD49+) population, but increased along a luminal differentiation route. MiR-34a depletion increased TEB size through an increase in the progenitor cell population [45]. Based on the effect of miR-34a on TEB development, it could be predicted that miR-34a confers a tumour-suppressive effect through the inhibition of a highly proliferative, cancer stem-cell-like phenotype. In several studies, miR-34a has been found to reduce cancer stemness by targeting genes such as CD24, NOTCH1, NOTCH4, HDAC1, or HDAC7. It has also been found to suppress tumour proliferation, EMT markers, and reduce chemoresistance.

Changes during pregnancy may result in a marked reduction in the proliferative population of cells in the mammary gland, leading to reduced breast cancer risk. In a comparison of Holstein cows during mid-pregnancy and during mid-lactation, miR-139 was found to be upregulated in pregnancy [46]. During pregnancy, there is a population of luminal progenitor cells that gradually differentiates as pregnancy advances, ending with fully differentiated cells in lactation [12]. MiR-139 mimics downregulated members of the IGF1R and PI3K/Akt pathways through binding of the *GHR* 3′UTR. In breast cancer, miR-139 reduced stemness through modulation of the PI3K/Akt pathway by targeting *CXCR4*. In vivo, miR-139 transfection in MDA-MB-231 cells reduced lung metastatic nodule development. Thus, miRNAs upregulated in pregnancy compared to lactation may be involved in directing the fate of progenitor cells.

For the maintenance of the progenitor population in the mammary gland, miR-205 is required. MiR-205 is expressed predominantly in basal cells through all development stages. It is also expressed in the mammary stem-cell population, although expression is markedly reduced mid-to-late lactation and in involution. In histological sections at the involution of the mammary gland, miR-205 expression was not detectable until the third day of involution with the return of the increased progenitor population. Transplantation of miR-205-deficient mammary epithelial cells revealed that miR-205 is not necessary for mammary gland development but supports stem cell regenerative potential [47]. In a review of miR-205 in breast cancer, its expression has been shown to decrease as breast cancer aggressiveness increases [48]. Indeed, in xenograft mouse models, miR-205 has been shown to reduce tumour growth and vasculogenic recruitment, characteristics of aggressive cancers closer to a stem-like phenotype. Thus, miRNAs enriched in the healthy progenitor cell population may become dysregulated in aggressive breast cancers, imparting a stem-like phenotype.

### 5.3. Epigenetic Regulation

Another mechanism by which cell lineages become restricted, such as from embryonic stem cells to a luminal cell in the developed mammary gland, is through epigenetic regulation. There are two types of epigenetic regulation commonly seen in the mammary gland: DNA methylation and histone modification. Hypomethylation is more commonly found in stem and progenitor cells, and methylation can direct cells into specialized identities. Most studies regarding epigenetic regulation in mammary gland development relate to silencing or activation of genes for lineage-specific differentiation such as luminal-driving *GATA3* or stem and basal-driving *Angptl2*. The role of epigenetic regulation in mammary gland development has been reviewed by Holliday et al. [49]. Although epigenetic regulation in mammary gland development is mainly studied on the coding gene level, miRNAs can promote or be regulated by regulators of methylation or histone modification. For example, the oncogenic miR-150, which is more highly expressed in pregnancy compared to lactation, has been found to repress members of the DNA methyltransferase family *DNMT3A* and *DNMT3B*, leading to an increase in the stem-cell-like population, likely due to hypomethylation [50]. Conversely, the puberty-related tumour suppressor miR-184 has been found to be methylated in lymph node metastases samples compared to normal tissue [51]. In puberty, miR-184 is found to be more highly expressed in ductal cells compared to the highly proliferative TEBs and may be identified as an anti-proliferative miRNA. Thus, the function of miRNAs found to be methylated in breast cancer can be examined through the lens of mammary gland development.

### 5.4. Invasion, Migration, and Proliferation

Invasion, migration, and proliferation are regulated through signaling pathways, including the PI3K/Akt/mTOR and Wnt/β-catenin pathways [52,53]. In puberty, the PI3K/Akt pathway can be activated by ligand-induced phosphorylation of fibroblast growth factor receptors or epidermal growth factor receptors, leading to proliferation and cell survival [13]. This pathway is dysregulated in breast cancer, leading to changes in cellular phenotype, metastasis, and drug resistance [52,53]. Dysregulated signaling pathways in puberty and cancer are regulated via miRNA. For example, miR-184 and miR-34a regulate the expression of genes and proteins of the phosphatidylinositol-3-kinase/protein kinase B (PI3K/Akt) and Wnt pathways, respectively. MiR-184 expression is increased in mature ducts compared to TEBs and reduces the activation of the PI3K/Akt by decreasing phosphorylation of Akt and related genes such as *AKT2*, *PRAS40*, and *GSK3A*. Through deregulation of these genes, tumour proliferation, invasion, and metastatic burden were reduced [51,54]. Similarly, miR-34a was found to reduce Wnt/β-catenin signaling in both puberty and breast cancer, regulating differentiation and suppressing stem-cell like characteristics [45].

During pregnancy, the mammary gland undergoes its final stage of development, once again characterized by significant proliferation and differentiation, leading to the expanded ductal structure that is capable of milk production. The highly proliferative cap cells of the TEB differentiate into myoepithelial cells. Basal and luminal progenitors differentiate into alveolar and ductal cells in pregnancy, which expand and invade the mammary fat pad [13]. The miR-17/92 cluster and miR-21 are both increased during pregnancy relative to early adulthood, indicating it may be involved in the cellular invasion or proliferation requisite for ductal structure growth [55,56]. In breast cancer, the miR-17/92 cluster promotes invasion and metastasis by targeting *HBP1*, the deactivation of which activates the Wnt/β-catenin pathway. By inhibiting miR-17 in vivo, metastasis of MDA-MB-231 cells was reduced by 50% [57]. In breast cancer, miR-21 is a key miRNA in the promotion of proliferation and dysregulation of apoptosis by inhibiting genes such as *PTEN, SMAD7,* and *PDCD4*, ultimately leading to deregulation of the PI3K/Akt/mTOR pathway [58,59]. MiR-21 in MCF-7 cells has also been shown to be modulated by exposure to alpha-linoleic acid in a time-dependent manner, reducing cell viability after 48 h [60]. It would be important to understand if alteration of miRNA regulating invasion, migration, or proliferation pathways during development may be a breast cancer preventative strategy. As well, it would be important to understand if shared miRNAs are altered similarly by dietary or drug interventions during puberty and during breast cancer.

### 5.5. Angiogenesis

During development, angiogenesis is most prominent during pregnancy and lactation during the rapid expansion of the ductal tree. Angiogenesis is necessary for alveolar development and facilitates optimal milk development. In human breast milk, there are high concentrations of vascular endothelial growth factor (VEGF). VEGF is secreted by mammary epithelial cells and mediates vascular growth and permeability during pregnancy and lactation [61]. VEGF has also been implicated in increased angiogenesis in breast cancer and is expressed by tumour endothelial cells. In breast cancer, increased angiogenesis provides nutrients and a platform for migration, leading to tumour growth and metastasis [62]. We found two miRNAs, miR-34 and miR-193b, to be involved in the regulation of angiogenesis in both pubertal and cancer processes. MiR-34 is a tumour suppressive miRNA which has been found to reduce vasculogenic mimicry in breast cancer by targeting AXL tyrosine kinase [63]. Overexpression of miR-34a was also found to reduce invasion and migration. As mentioned previously, miR-34a inhibition led to increased TEB size, an increased progenitor pool, and larger mammary gland in puberty. In puberty, recruitment of vasculature accompanies the rapid proliferation of the TEB. The anti-proliferative effect of miR-34a in puberty corresponds to its anti-angiogenic effect in breast cancer. However, we found no studies confirming that increased angiogenesis contributes to miR-34a-mediated proliferation in puberty.

In pregnancy, miR-193b deletion increased luminal differentiation and proliferation in non-parous and pregnant mice. Yoo et al. postulate that miR-193 mediates proliferation during puberty and pregnancy under the cytokine induced transcription factor STAT5 [64]. STAT5 and prolactin, a key hormone during pregnancy and lactation, have been implicated in a positive autocrine feedback loop which promotes angiogenesis [65]. Thus, the anti-proliferative role of miR-193b is in line with its characterization as a tumour suppressor in breast cancer (Table 1). Mir-193b has been found to reduce vasculogenic mimicry in MDA-MB-231 cells, a triple-negative breast cancer cell line [66]. Other tumour suppressive roles of miR-193 are a reduction in metastasis and drug resistance, which may be mediated by its anti-angiogenic role. Angiogenesis in puberty and pregnancy is partially regulated through miRNA expression, and these miRNAs can allow for identification and further understanding of angiogenic mechanisms which promote metastasis and invasion in breast cancer.

### 5.6. Apoptosis

Apoptosis is an important process for normal breast development. When apoptotic processes are disrupted and reduced, breast cancer arises [67]. Involution is triggered by sustained weaning and returns the mammary gland from a lactating state to its pre-pregnancy state. It is characterized by a significant increase in apoptosis, breaking down of the basement membrane, expression of metalloproteinases, and recruitment of phagocytes [68]. During involution, the mammary gland regains its pre-pregnancy potential for lactation, including having a carried over and likely newly generated alveolar progenitor population [40]. One miRNA more highly expressed in involution compared to lactation and pregnancy is miR-142-3p. MiR-142-3p targets the 3′ UTR of the prolactin receptor (*PRLR*) mRNA transcript. *PRLR* is required for the function of prolactin in lobuloalveolar maturation and milk synthesis. MiR-142-3p overexpression increased apoptosis and decreased proliferation in primary murine mammary gland epithelial cells. Downstream, signaling pathways downregulated by miR-142-3p included the apoptosis/protein synthesis-regulating Janus kinase/signal transducer and activator of transcription protein (JAK/STAT) and proliferation-regulating MAPK pathways [69]. In breast cancer, miR-142-3p has a largely tumour-suppressive, anti-proliferative effect (Table 1), increasing apoptotic markers such as the caspase family of cysteine proteases [70,71,72]. Similarly, the miR-424(322)/503 family increases in involution, and knockout of this miRNA reduces acini destruction and apoptosis [73]. Thus, the miR-424(322)/503 family plays an important role in apoptosis and has a tumour-suppressive effect in breast cancer (Table 1). MiR-424-5p has been shown to reduce chemoresistance and decrease breast cancer proliferation by inducing apoptosis and targeting the PI3K/Akt/mTOR pathway [74,75]. By investigating the role of miRNAs in apoptosis, particularly in involution, future studies may identify dysregulated apoptotic miRNAs as potential therapeutic targets.

**Table 1:** MicroRNAs found to be involved in both mammary gland development and breast cancer.

MiRNA	Development		Breast Cancer ^1^	
	Population Characteristics	Outcomes	Population Characteristics	Outcomes
***Embryo stage***				
miR-137	Embryos from ICR (CD-1) time-mated pregnant mice [34]	- miR-137 was increased in the embryonic mammary gland compared to surrounding region - ↑ miR-137 → ↑ epithelium thickness, failure to invade underlying mesenchyme	Ex vivo tissue, breast cancer cell lines	*Oncogenic* ↑ EMT, invasion [76] *Tumour suppressive* ↓ tumour weight, volume, invasion, proliferation, migration, EMT, drug resistance, stemness [34,36,37,38,77,78,79,80]
miR-206	Embryos from ICR time-mated pregnant mice [41]	- ↑ miR-206 in the dermal and mammary mesenchyme at E11.5-E13.5 and fat pad-forming layers - ↑ miR-206 → ↓ ER-α, *Tachykinin1*, *Lef1*, *Gata3* (eliminated in mesenchyme); ↑ *Tbx3* (mesenchyme)	Ex vivo tissue, breast cancer cell lines	*Oncogenic* ↑ migration, invasion, proliferation [44] *Tumour suppressive* ↓ proliferation, drug resistance, metastasis, stemness ↑ apoptosis [27,42,43,81,82] ^2^
***Puberty stage***				
miR-184	5-week-old β-actin-GFP reporter FVB/n mice [51]	- ↑ miR-184 in mature ducts vs. TEBs - ↑ miR-184 in differentiation/proliferation/invasion of TEBs into ductal epithelial cells	Ex vivo tissue, breast cancer cell lines, mouse tumour models	*Tumour suppressive* ↓ proliferation, invasion, methylation, metastasis ↑ cell cycle arrest [51,54,83]
miR-34a	miR-34-knockout C57BL/6J (Trp53 strain) mice [45]	- ↓ miR-34a → ↑ TEB size	Ex vivo tissue, breast cancer cell lines, mouse tumour models, review	*Tumour suppressive* ↓ stemness, invasion, migration, tumour volume and growth, EMT, proliferation, drug resistance ↑ apoptosis, cell cycle arrest [27,51,54,63,83,84,85,86,87,88,89,90,91,92,93,94,95] ^2^
miR-489	6-week-old FVP mice [96]	- ↑ miR-489 in stem-like cells vs. luminal, luminal-progenitor, and myoepithelial cells - ↑ miR-489 at 7 weeks vs. lactation day 9 and involution	Ex vivo tissue, breast cancer cell lines, mouse tumour models	*Tumour suppressive* ↓ proliferation, migration, invasion, drug resistance, stemness, tumour volume ↑ apoptosis, sensitivity to drugs [96,97,98,99,100,101,102,103,104,105]
	4- and 6-week-old MMTV-miR-489 mice (*n* = 9)	- ↓ ductal growth, TEB formation, Ki-67+ cells		
***Virgin adult and pregnancy***				
miR-17/92 cluster	miR-17-92b^fl/fl;MMTV-Cre^ mice [55]	- ↑ miR-17/92 (2–3.5x) pregnancy day 6 vs. virgin adult - miR-17/92 deletion did not affect pubertal development or lactation	Review	*Oncogenic* ↑ proliferation, migration, invasion, angiogenesis, metastasis, chemoresistance [28,58,106] ^2^
miR-193b	C57BL/6 miR-193b^−/−^ mice [64]	- miR-193b deletion → ↑ differentiation in non-parous and pregnancy	Ex vivo tissue, breast cancer cell lines	*Tumour suppressive* ↓ growth, metastasis, migration, invasion, stemness, chemoresistance ↑ apoptosis [66,107,108,109,110,111,112,113]
miR-21	Stat5^fl/fl;Cre^ mice, miR-21^−/−^ mice [56]	- ↑ prolactin → ↑ miR-21 (HC-11 cells) - ↑miR-21 pregnancy day 6 vs. virgin adult (↑ proliferation) - ↓ STAT5 → ↓ miR-21 - miR-21 dispensable for mammary gland development	Review	*Oncogenic* ↑ invasion, migration, proliferation, metastasis, radiotherapy and chemoresistance, tumour growth ↓ apoptosis [58,59,114,115,116,117] ^2^
***Pregnancy and lactation***				
miR-27a	Three-year-old Xinong Saanen Dairy Goat (*n* = 3) [118]	- ↑ miR-27a mid-lactation vs. dry period (pregnancy/involution) - ↑ miR-27a → ↓ triglyceride accumulation in cells, ↓ unsaturated:saturated fatty acid ratio	Ex vivo tissue, breast cancer cell lines, review	*Oncogenic* ↑ cell growth, EMT, demethylation of tumour suppressor ↓ apoptosis [81,114,119] ^2^
miR-139	Holstein cows mid-pregnancy (*n* = 3), mid-lactation (*n* = 3, 90 days in milk) [46]	- - ↑ miR-139 in pregnancy vs. mid-lactation - - ↑ miR-139 ⊣ β-casein → ↓ p-Stat5, IGF1R, p-AKT1, AKT1, Cyclin D1 (IGF1R and GHR signaling pathway)	Ex vivo tissue, breast cancer cell lines, mouse tumour models	*Oncogenic* ↓ apoptosis [120] *Tumour suppressive* ↓ proliferation, migration, invasion, EMT, stemness ↑ apoptosis [121,122,123,124,125,126,127,128,129,130]
miR-150-5p	Stop-150^fl/^fl C57BL/6 mice [131]	- ↑ miR-150-5p in pregnancy day 14 vs. lactation day 2 - ↑ miR-150-5p → ↓ *FASN*, ACACA, OLAH - ↑ miR-150-5p → ↓ de novo fatty acid synthesis		*Oncogenic* ↑ cell proliferation, drug resistance, migration, EMT, stem-like characteristics [50,132,133]
miR-204-5p	Pregnant and lactating C57BL/6J mice (*n* = 6 per group) [134]	- ↓ miR-204-5p in pregnant vs. lactating mice	Ex vivo tissue, breast cancer cell lines	[27,135,136,137,138] ^2^
	HC11 cells	- ↑ miR-204-5p → ↑ casein, milk lipid synthesis through *SIRT1*		
miR-206	Mammary gland from 2- month adult, pregnancy day 10, and lactation day 6 [42]	- ↑ in pregnancy vs. virgin and lactation - miR-206 → G1-S cell cycle arrest, ↓ stemness markers (HC11)	Ex vivo tissue, breast cancer cell lines	*Oncogenic* ↑ migration, invasion, proliferation [44] *Tumour suppressive* ↓ proliferation, drug resistance, metastasis, stemness ↑ apoptosis [27,42,43,81,82] ^2^
miR-486	Multiparous Holstein cows in high-quality lactation (*n* = 3), low-quality lactation (*n* = 3), and pregnancy (*n* = 3) [139]	- ↓ miR-486 in pregnancy vs. lactation	Ex vivo tissue, breast cancer cell lines	*Tumour suppressive* ↓ invasion, migration, stemness, proliferation, EMT ↑ apoptosis, radiosensitivity, chemosensitivity, cell cycle arrest [140,141,142,143]
	Bovine mammary epithelial cells	- miR-486 ⊣ PTEN - miR-486 → ↑ Akt, mTOR - miR-486 → ↑ β-casein, lactose, triglyceride secretion		
***Pregnancy, lactation and involution***				
miR-103	30 healthy three-year-old Xinong Saanen dairy goats mid-lactation (120 days after parturition) and dry lactation (60 days before parturition) [144]	- ↑ miR-103 mid-lactation vs. parturition/involution/pregnancy - ↑ miR-103 promotes milk fat droplet, triglyceride accumulation in in goat mammary epithelial cells	Ex vivo tissue, breast cancer cell lines	*Oncogenic* ↑ metastasis, EMT [145,146]
miR-152	Mammary gland from Han ewes (*n* = 3) Day −8, −6, −4, −1 from parturition (involution), and 1 week after parturition [147]	- ↑ miR-152 in pregnancy/involution vs. lactation	Ex vivo tissue, breast cancer cell lines, mouse tumour models	*Tumour suppressive* ↓ proliferation, migration, invasion, cell survival, EMT, stemness, methylation, chemotherapy resistance, metastasis ↑ apoptosis, cell cycle arrest [148,149,150,151,152,153,154,155,156,157]
miR-218	Mammary gland from Han ewes (*n* = 3) Day −8, −6, −4, −1 from parturition (involution), and 1 week after parturition [147]	- ↑ miR-218 in pregnancy/involution vs. lactation	Ex vivo tissue, breast cancer cell lines	*Oncogenic* ↑ metastasis, invasion, migration, EMT, methylation [158,159,160,161,162] *Tumour suppressive* ↓ proliferation, migration, chemoresistance, invasion ↑ apoptosis [163,164,165,166,167,168]
miR-223	FVB MMTV-Δ16HER2 miR-223 knockout mice [169]	- inverse correlation between miR-223 and developmental stage, lowest day after parturition	Breast cancer cell lines, review	*Oncogenic* ↑ EMT, metastasis, drug resistance [170] *Tumour suppressive* ↓ drug resistance, proliferation, migration, EMT ↑ apoptosis [169,171,172]
miR-31	TRE-miR-31 transgenic mice from C57BL/6J background [173]	- miR-31 knockout ↑ alveolar differentiation, ↓ proliferation in TEB - miR-31 knockout mice gave birth but were unable to nurse pups due to undifferentiated ductal structures and failure to form alveoli in pregnancy	Breast cancer cell lines	*Tumour suppressive* ↓ invasion, migration, proliferation ↑ apoptosis, chemotherapy sensitivity [27,174,175,176] ^2^
***Lactation***				
miR-148a	Three-year-old Xinong Saanen dairy goats non-pregnant, early-lactation, peak-lactation, late-lactation (15, 60, 150 days after parturition), and dry-lactation [177]	- ↑ miR-148 in lactation - ↑ miR-148 ↑ triglyceride and cholesterol in goat mammary epithelial cells	Breast cancer cell lines, mouse tumour models	*Oncogenic* Inhibition led to ↓ proliferation [178] *Tumour suppressive* ↓ proliferation, metastasis, chemoresistance, stemness ↑ apoptosis [27,179,180,181,182,183,184] ^2^
miR-17-5p	Three-year-old Xinong Saanen dairy goats non-pregnant, early-lactation, peak-lactation, late-lactation (15, 60, 150 days after parturition), and dry-lactation [177]	- ↑ miR-17-5p in lactation - ↑ miR-17-5p ↑ triglyceride and cholesterol in goat mammary epithelial cells	Ex vivo tissue, breast cancer cell lines, mouse tumour models	*Tumour suppressive* ↓ cell proliferation ↑ apoptosis [185,186] *Oncogenic* ↑ migration, invasion, proliferation, cell growth, angiogenesis, metastasis [58,187,188] ^2^
miR-181b	Three-year-old Xinong Saanen dairy goats non-pregnant, early-lactation, peak-lactation, late-lactation (15, 60, 150 days after parturition), and dry-lactation [189]	- ↑ miR-181b in dry-lactation compared to non-pregnant, lowest during peak lactation - ↑ miR-181b ↓ triglyceride and cholesterol	Breast cancer cell lines, review	*Tumour suppressive* ↓ cell proliferation, migration, invasion ↑ apoptosis [190] *Oncogenic* ↑ migration, proliferation, chemoresistance, cell cycle, EMT [58] ^2^
miR-25	Three-year-old Xinong Saanen dairy goats non-pregnant, early-lactation, peak-lactation, late-lactation (15, 60, 120 days after parturition), and dry-lactation [191]	- ↑ miR-25 in non-pregnant, ↓ during lactation - ↑ miR-25, ↓ triglyceride, lipid-droplets	Ex vivo tissue, breast cancer cell lines, tumour mouse models	*Oncogenic* ↓ apoptosis ↑ migration, invasion, proliferation, chemoresistance, EMT, tumour volume [192,193,194,195,196,197]
***Involution***				
miR-424(322)/503	miR-424(322) and miR-503 knockout mice [73]	- ↑ miR-424(303)/503 in involution - miR-424(322)/503 knockout presented reduced acini destruction and apoptosis in involution - miR-424(322)/503 ⊣ BCL-2, IGF1R	Ex vivo tissue, breast cancer cell lines, tumour mouse models	*Tumour suppressive* ↓ migration, drug resistance, invasion, tumorigenesis, EMT, stemness, invasion, tumour growth ↑ apoptosis, cell cycle arrest [27,74,75,198,199,200,201,202,203,204,205] ^2^
***Virgin adult, pregnancy, lactation and involution***				
miR-126	Mouse (strain not specified) at virgin, pregnancy day 5, lactation day 0, lactation day 5, lactation day 10, involution day 10 [206]	- ↑ miR-126 in all lactation days vs. virgin, pregnancy, involution - ↓ miR-126 ↑ lipid metabolism - estradiol and progesterone reduced miR-126-3p expression	Ex vivo tissue, breast cancer cell lines, tumour mouse models	*Tumour suppressive* ↓ metastasis, angiogenesis, cell growth, proliferation, EMT markers, migration, drug resistance ↑ cell cycle arrest [207,208,209,210,211,212,213,214,215,216,217,218,219]
miR-126-3p	Female BALB/C mice mammary tissue from virgin, pregnancy, lactation, and involution at 3 time points within each (*n* = 1/time point) [220]	- ↓ miR-126-5p in lactation and pregnancy vs. virgin and involution - miR-126-5p ⊣ *Pgr*	Breast cancer cell lines	*Tumour suppressive* ↓ invasion, migration [210,221]
miR-142-3p	Female BALB/c mice mammary tissue from virgin 4, 5, 7 weeks, pregnancy 5, 13, 18 days, lactation 3, 7, 13 days, involution 2, 5, 10 days [69]	- ↓ miR-142-3p in lactation and pregnancy vs. virgin and involution - ↑ miR-142-3p in involution vs. lactation - ↓ miR-142-3p in involution vs. virgin - miR-126-5p ⊣ *Prlr* → ↓ Akt/mTOR, MAPK, STAT5	Ex vivo tissue, breast cancer cell lines, tumour mouse models	*Tumour suppressive* ↓ invasion, migration, proliferation, chemoresistance, cell size, cell volume, EMT, metastasis ↑ apoptosis, cell cycle arrest [222,223,224] [70,71,72,225,226,227,228,229] *Oncogenic* ↑ metastasis [230]
miR-15b	Mice ^2^ mammary gland from mature virgin (8 weeks), pregnancy day 5, lactation day 0, lactation day 5, lactation day 10 [231]	- miR-15b virgin > pregnancy day 5 > lactation day 0 = lactation day 5 = involution day 10 > lactation day 10 - estradiol and progesterone together reduce miR-15b levels (MCF-10A cells) - ↑ miR-15b, ↓ lipid metabolism	Ex vivo tissue, breast cancer cell lines	*Oncogenic* ↓ apoptosis ↑ migration, invasion, cell size, cell volume, proliferation [232,233,234]
miR-205	miR-205-lacZ and miR-205^fl/fl^ mice from C57BL6/129s mixed background [47]	- ↑ miR-205 in mammary basal and stem cells (high expression at TEB caps) of across all development stages, highest during lactation - no miR-205 expression in alveolar structures - miR-205 knockout reduced basal cell population and reduced collagen deposition regulating YAP and Wnt	Breast cancer cell lines, tumour mouse models	*Tumour suppressive* ↓ proliferation, migration, invasion, EMT, angiogenesis, radio/chemotherapy resistance ↑ apoptosis [27,48,81,114,235,236,237,238,239] ^2^
	SCID/beige mice transplanted with miR-205^fl/fl^ mammary cells	- ↓ miR-205, ↓ mammary ductal structures		
miR-206	MMTV-Cre Brca1^Co/Co^ mice [42]	- miR-206 in virgin > involution = mid-pregnancy > lactation - ↓ Brca1→ ↑ miR-206	Ex vivo tissue, breast cancer cell lines	*Oncogenic* ↑ migration, invasion, proliferation [44] *Tumour suppressive* ↓ proliferation, drug resistance, metastasis, stemness ↑ apoptosis [27,42,43,81,82] ^2^
	MMTV miR-206 mice of FVB/NJ background [240]	- MMTV miR-206 glands ↓ ductal and end bud structures		
miR-221	Mice ^3^ mammary gland from mature virgin (8 weeks), pregnancy day 5, lactation day 0, lactation day 5, lactation day 10, involution day 10 [241]	- miR-221 in virgin > pregnancy day 5 > involution day 10 > lactation day 0 = lactation day 5 > lactation day 10 - miR-221 reduces lipid metabolism (MCF-10A cells) - estradiol and progesterone together reduce miR-221 levels (MCF-10A cells)	Review	*Oncogenic* ↓ apoptosis ↑ drug resistance, EMT, proliferation, metastasis, invasion [58,114,242] ^2^
miR-30b	MMTV-LTR miR-30b transgenic mice of FVB/N background [243]	- miR-30b in virgin > puberty, pregnancy day 18 > pregnancy day 12, lactation day 3 = lactation day 10 > involution day 3 - no impact of miR-30b overexpression in virgin and gestating mice - miR-30b ↓ mammary acini in lactation, fewer lipid droplets, but irregularly shaped → impaired growth in pups (non-lethal) - ↑ miR-30b → delay in involution day 3 and 6 post-weaning	Ex vivo tissue, breast cancer cell lines, review	*Tumour suppressive* ↑ chemotherapy sensitivity, cell cycle arrest [27] ^2^ *Oncogenic* ↑ proliferation, migration, invasion [244]

^1^ Expanded table with population details can be found in Supplementary Table S1. ^2^ Review paper(s) included largely indicate oncogenic/tumour suppressive phenotype. ^3^ Type not specified.

## 6. Conclusions and Future Directions

Since 2006, there have been major strides in understanding the diverse and necessary role of miRNAs as regulators in all biological processes, including mammary gland development. There are clear parallels between processes regulated by miRNAs involved in mammary gland development and breast cancer, with 32 miRNAs so far identified to contribute to both. MiRNAs that stray from their role in development and homeostasis contribute to the development, severity, and prognosis in breast cancer. Processes found to be present in development and aberrant in cancer include EMT, invasion, migration, proliferation, epigenetic regulation, apoptosis, and characteristics such as stemness. By examining miRNAs in development, the mechanisms behind breast cancer incidence, severity, and metastasis can be explained or predicted. From the embryo, miRNAs can be examined for their role in EMT and stemness. During puberty and pregnancy, miRNAs involved in invasion, proliferation, and migration can be investigated. Similarly, the processes of invasion, proliferation, and differentiation can be examined in pregnancy. After lactation, miRNAs involved in the massive apoptosis of the lactation structures can give clues to regulators of apoptosis in breast cancer. Dysregulation of miRNAs in any of these processes can lead to incidence or increased risk of breast cancer. Furthermore, examining miRNAs modified during developmental stages may help to identify and improve the understanding of the function of miRNAs dysregulated in breast cancer. In fact, miRNAs found in development contributing to processes known to be dysregulated in breast cancer can be further investigated.

While studying effect of individual or paired miRNAs in vitro or in vivo is most common, the synergistic effects of miRNA are under explored in breast cancer. During development or homeostasis, miRNAs work in concert to coordinate cellular activity. During mammary gland development, distinct temporal miRNA expression patterns are found during each stage. Further study is required to determine the importance of miRNA synergy during development and their role in breast cancer.

Over a lifetime, miRNAs, genes, and proteins act in concert to regulate development and homeostasis. Understanding these functions are important to better understand how malignancies can arise from their dysregulation. This is especially true for breast cancer, a heterogenous disease which requires consideration of its diverse phenotypes in developing treatments. Since miRNAs can alter mammary gland morphology and breast cancer characteristics, they have immense potential to be nutritional or drug targets for the prevention or treatment of breast cancer.

## 7. Methods

### 7.1. Mammary Gland Development miRNA Search Strategy

To find miRNAs involved in mammary gland development, PubMed was searched for all original research articles for mammary gland development including animal and human studies. The following search terms were used: “((mammary development) OR (mammary gland development) OR (breast development)) AND (miRNA OR microRNA) NOT (breast cancer)[Title] NOT (tumor[Title]) NOT (carcinoma[Title])”. Articles were screened using the Population, Interventions, Comparisons, Outcomes, and Study Designs (PICOS) [245] elements, as outlined in Table 2. Eligibility criteria included primary animal and human studies comparing miRNA expression in 2 or more developmental stages or elucidation of miRNA function in ≥1 developmental stage. Studies examining cell lines, serum, blood, or milk miRNAs were excluded. Data extracted included strain/type/age of animal or human, miRNAs investigated, and the effect of the miRNA on developmental structures or stages.

### 7.2. Breast Cancer miRNA Search Strategy

MiRNAs identified in Section 7.1. (identified as *x*) were searched in PubMed using the search query “miR-*x*” AND “breast cancer”. The PICOS search strategy is outlined in Table 3. Articles were excluded if only using computational inference.

## Figures and Tables

**Table 2:** Mammary gland development PICOS search strategy as applied in this review.

PICOS Component	Search Strategy/Terms
Population	((mammary development) OR (mammary gland development) OR (breast development))
Intervention	(miRNA OR microRNA)
Comparisons	Mammary gland stages
Outcomes	Changes in miRNA expression, miRNA functional analysis in mammary gland
Study design	Primary research in animal models, human studies

**Table 3:** Breast cancer PICOS search strategy as applied in this review.

PICOS Component	Search Strategy/Terms
Population	Breast cancer cell lines, xenografts, mouse models, biopsies
Intervention	miR-*x*
Comparisons	Normal/healthy/non-cancerous mammary cells/tissue vs. cancerous OR Non-metastatic cells/tissue vs. metastatic OR Treatment resistant cells/tissue vs. non-treatment resistant
Outcomes	Breast cancer occurrence, severity, metastatic potential, treatment resistance
Study design	Primary research and reviews on miRNAs in breast cancer cell lines, murine models, human studies

## Data Availability

Not applicable.

## Supplementary Materials

The following supporting information can be downloaded at: https://www.mdpi.com/article/10.3390/ijms232415978/s1.
